# Circulating tumor cell detection in hepatocellular carcinoma based on karyoplasmic ratios using imaging flow cytometry

**DOI:** 10.1038/srep39808

**Published:** 2016-12-23

**Authors:** Zixin Liu, Weixing Guo, Dandan Zhang, Yanan Pang, Jie Shi, Siqin Wan, Kai Cheng, Jiaqi Wang, Shuqun Cheng

**Affiliations:** 1 Eastern Hepatobiliary Surgery Hospital (EHBH), the Second Military Medical University, Shanghai 200433, China; 2 Clinical Research Center, Changhai Hospital, Second Military Medical University, Shanghai 200433, China

## Abstract

Circulating tumor cells (CTCs) originate from tumor tissues and are associated with cancer prognosis. However, existing technologies for CTC detection are limited owing to a lack of specific or accurate biomarkers. Here, we developed a new method for CTC detection based on the karyoplasmic ratio, without biomarkers. Consecutive patients with liver cancer or non-cancer liver diseases were recruited. CTCs in blood samples were analyzed by imaging flow cytometry based on the karyoplasmic ratio as well as EpCAM and CD45. Microvascular invasion (MVI), tumor recurrence, and survival were recorded for all patients. A total of 56.2 ± 23.8/100,000 cells with high karyoplasmic ratios (HKR cells) were detected in cancer patients, which was higher than the number of HKR cells in the non-cancer group (7.6 ± 2.2/100,000). There was also a difference in HKR cells between liver cancer patients with and without MVI. Based on a receiver operating characteristic curve analysis, the threshold was 21.8 HKR cells per 100,000 peripheral blood mononuclear cells, and the area under the curve was higher than those of traditional methods (e.g., CD45 and EpCAM staining). These results indicate that the new CTC detection method was more sensitive and reliable than existing methods. Accordingly, it may improve clinical CTC detection.

Circulating tumor cells (CTCs) originate from tumor tissues and are released into the peripheral blood^1^. Several studies have indicated that CTCs are an independent risk factor associated with the prognosis of solid tumors, such as breast cancer, colon cancer, prostate cancer, and hepatocellular carcinoma (HCC)^2^. CTCs may be an active source of HCC metastasis or recurrence. Patients with higher CTC counts may have poorer outcomes, higher recurrence risks, and lower disease-free survival and overall survival after surgery^3^. Accordingly, a variety of methods have been developed to detect and analyze CTCs^4^. The direct analysis of unpurified nucleated cells from blood or diluted blood samples by tumor-specific staining is simple, but has limited applications and stability because typical biomarkers, e.g., CD133 and EpCAM, are only expressed in a small fraction of CTCs^5^,^6^. Bulk blood-processing methods, such as flow cytometry and magnetophoresis, have a tendency to exclude rare cells, but are popular for CTC detection owing to their use of simple and readily available tools. The morphological properties that are shared by all tumor cells, such as size, deformability, and density, can be applied for CTC detection^1^,^7^.

We developed and validated an imaging flow cytometry assay to quantify CTCs based on the nuclear-cytoplasmic ratio in peripheral blood samples. This method greatly increased the sensitivity of CTC detection. In HCC patients, the number of CTCs is associated with the presence of microvascular invasion (MVI)^8^. Owing to the lack of specific biomarkers and the high cost, current CTC detection methods are not appropriate for clinical application^5^. Our method does not rely on biological agents, such as antibodies, enabling a faster assay with high stability. Interestingly, we found a strong association between CTC counts and the karyoplasmic ratio, the presence of MVI, and the prognosis of HCC.

## Results

### Development of a new CTC detection assay based on a high karyoplasmic ratio

**Figure 1:**
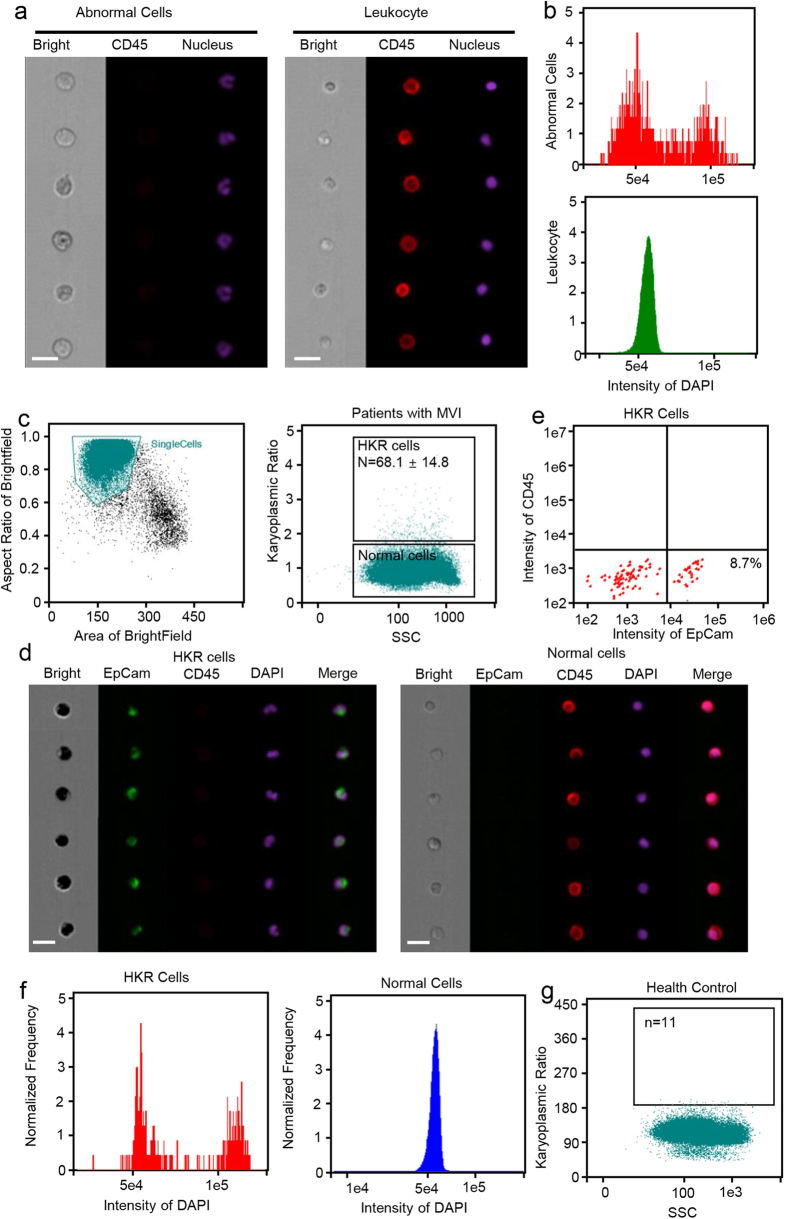
Detection of cells with abnormal nuclei using imaging flow cytometry. (**a**) Representative cell images with the same DAPI intensity. CD45, a lymphocyte biomarker, was labeled with PE-Cy5. Upper panel: CD45^+^ lymphocyte with a smaller DAPI area. Lower panel: CD45^−^ cells with a larger DAPI area, showing a looser structure of nuclei. Scale bars represent 20 μm. (**b**) Mean DAPI fluorescence intensity in PBMCs. Unlike the cells with a normal DAPI area (lower panel), a G1 and G2 peak could be detected in the cells with larger DAPI areas (upper panel). (**c**) Imaging flow cytometry test results for the peripheral blood samples from MVI patients. Left panel, basic results for peripheral blood mononuclear cells (PBMCs), the horizontal axis indicates the cell area and the vertical axis indicates the aspect ratio. The gate displays the group of single cells. Right panel: patients with MVI had 68.1 ± 14.8 cells with large DAPI areas. (**d**) Representative images for PBMCs from HCC patients. CD45, a lymphocyte biomarker, was labeled with PE-Cy5. EpCAM, a biomarker of circulating tumor cells (CTCs), was labeled with FITC. Left panel: cells with high karyoplasmic ratios (HKR cells), which were EpCAM-positive and CD45-negative. Right panel: cells with normal karyoplasmic ratios (normal cells), which were EpCAM-negative and CD45-positive. Scale bars represent 20 μm. (**e**) Flow cytometry test results for HKR cells from MVI patients. All HKR cells were CD45^−^, but only 8.7% of HKR cells were EpCAM^+^.(**f**) Mean DAPI fluorescence intensity in PBMCs. Unlike cells with normal DAPI areas (right panel), a G1 and G2 peak could be detected in the cells with larger DAPI areas. (**g**) Karyoplasmic ratio detection in PBMCs. Nuclei were marked using DAPI. Compared to the patients with MVI, normal individuals had more cells with large DAPI areas.

Cells with abnormal nuclei were found in blood samples from HCC patients using imaging flow cytometry. After DAPI staining and antibody labeling, a group of CD45^−^ cells with larger nuclei than those of CD45^+^ cells was found (Fig. 1a). When we analyzed this cell group separately, G1 and G2 peaks were observed, indicating the capacity for cell division (Fig. 1b). Normal peripheral blood nucleated cells are terminally differentiated and lack the capacity for cell division. Therefore, we deduced that these cells might be CTCs. However, in the peripheral blood samples, large nuclei were observed not only in tumor cells, but also in other cell types, such as exfoliated epithelial cells and adhesion cells (Supplementary Figure S1).

Ten samples from HCC patients with MVI were examined in more detail. The peripheral blood cells were marked with antibodies against CD45 and EpCAM as well as with DAPI and examined by imaging flow cytometry (Fig. 1d). A new parameter, i.e., the ratio of the area of the nucleus to that of the cytoplasm, was defined as the karyoplasmic ratio of cells (Supplementary Figure S2). Using this parameter, single nuclear cells of the peripheral blood could be divided into two categories (Fig. 1c). The group characterized by a low karyoplasmic ratio included 99.8% of total cells. Most cells in this group were CD45^+^EpCAM^−^ cells with no G2 peak (Fig. 1d and e) and were considered normal single nuclear peripheral blood cells. In the other group of cells, which had a higher karyoplasmic ratio (approximately 1.5 times to 2 times that of normal cells, Table 1), only 68.1 ± 14.8/100000 cells in peripheral blood samples were included (Table 1). However, 8.7% of cells in this group were CD45^−^EpCAM^+^ cells with significant G2 peaks (Fig. 1d,e and f). These results indicated that the cells with a higher karyoplasmic ratio were tumor cells with to ability to divide. These cells with high karyoplasmic ratios were named HKR cells.

To further study this group of cells, blood samples obtained from five healthy volunteers were randomly selected and tested following the same methods. Peripheral blood from normal individuals contained only 7.1 ± 2.2/100000 HKR cells (Table 2, Fig. 1g), which was far fewer than the number of HKR cells observed in patients with MVI (*P* = 0.00675). Some HKR cells detected in normal individuals were EpCAM^−^ and exhibited the same DAPI intensity as that of normal cells. These are likely terminally differentiated cells from normal circulating cells that lack the ability to undergo cell division.

These results indicated that most HKR cells detected using our method in peripheral blood samples obtained from HCC patients with MVI are CTCs, and the HKR cell counts differ significantly between samples obtained from healthy individuals and HCC patients.

### Confirmation of the HKR cell assay using clinical samples

**Figure 2:**
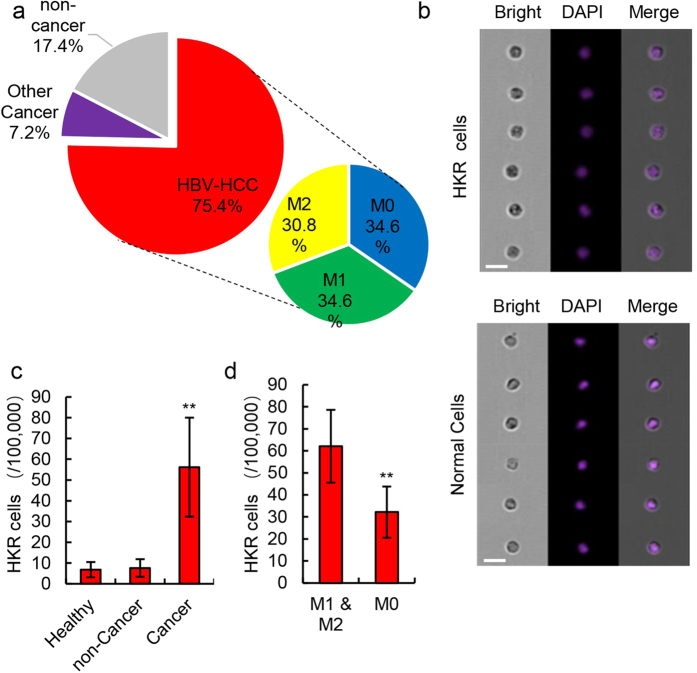
Differentiation of HCC based on HKR cell content. (**a**) The source or composition of samples. (**b**) Representative results for the *in vivo* cellular localization of DAPI (Ex: 405 nm, Em: 454 nm) in peripheral blood mononuclear cells analyzed by real-time imaging flow cytometry 1 h after DAPI staining. Two groups of cells with different nuclear morphology are shown. Scale bars represent 20 μm. (**c**) Number of HKR cells in 100,000 peripheral blood mononuclear cells for three different groups. HKR cells were identified by imaging flow cytometry. Data are presented as means ± SD of three independent experiments, **P < 0.01. (**d**) Number of HKR cells in 100,000 peripheral blood mononuclear cells in the no MVI group (M0) and the MVI group (M1 & M2). HKR cells were identified by imaging flow cytometry. Data are presented as means ± SD of three independent experiments, **P < 0.01.

We recruited 12 healthy volunteers, 52 HCC patients, 5 cholangiocarcinoma patients, and 12 patients with noncancerous liver diseases (Fig. 2a). Of the 52 HCC patients, 18 (34.6%) were MVI0, 18 (34.6%) were MVI1, and 16 (30.8%) were MVI2 (Fig. 2a). The noncancerous liver disease patients included liver cirrhosis after hepatitis B (n = 2), hepatic hemangioma (n = 2), choledochocyst (n = 2), focal nodular hyperplasia (n = 2), and hepatic calculus (n = 4) (Table 2). Other clinical features of healthy volunteers and patients are provided in Supplementary Table 1.

After examination by imaging flow cytometry, 6.8 ± 3.7/100,000 HKR cells were detected in 12 healthy peripheral blood samples and 7.6 ± 2.2/100,000 HKR cells were detected in 12 non-cancerous patient blood samples, and these numbers were similar to the number of HKR cells observed in normal volunteers (*P* > 0.05). In 57 cancer blood samples (including 52 HCC patients and 5 cholangiocarcinoma patients), 56.2 ± 23.8/100,000 HKR cells were detected, and this number was significantly different from those of normal blood samples and non-cancer blood samples (Table 2, Fig. 2b and c; *P* = 0.000538). Significant differences were also observed between blood samples obtained from HCC patients with MVI (M1 or M2, 62.0 ± 16.6/100,000 cells) and from HCC patients without MVI (M0) (Fig. 2d, 32.2 ± 11.6; *P* = 0.006157). No significant difference was found between HCC and cholangiocarcinoma samples (Supplementary Figure S3; *P* = 0.54437).

In addition, metastasis, organ invasion, and lymph node invasion were correlated with the number of cells with an abnormal nuclear-cytoplasmic ratio, while other indicators were not correlated. The correlations between abnormal nuclear-cytoplasmic ratio cell counts and various indicators are summarized in Table 3.

### The diagnostic value of the HKR cell assay and comparison with circulating tumor cell detection based on CD45 and EpCAM expression

**Figure 3:**
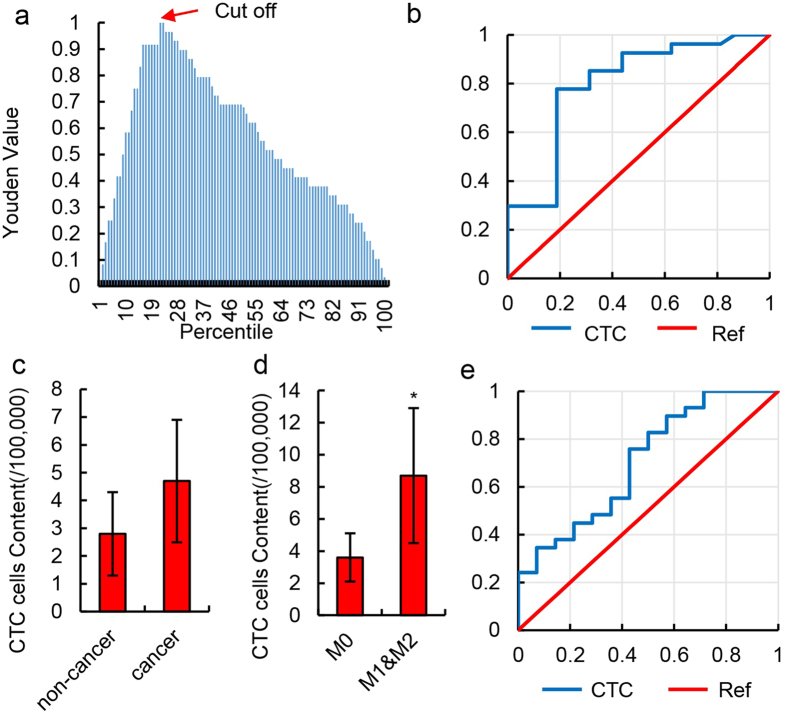
Diagnostic value of HKR cell content. (**a**) Youden curve for HKR cells in 52 HCC patients and 12 normal individuals. Arrow refers to the maximum Youden index, which was defined as the threshold. (**b**) ROC curve of HKR cells for 23 HCC patients with MVI (M1 & M2) and 18 HCC patients without MVI (M0). HKR cells were identified by imaging flow cytometry. (**c**) Relative number of CTCs in 100,000 peripheral blood mononuclear cells in non-cancer liver disease patients and HCC patients. CTCs were defined as CD45^−^ & EpCAM^+^ cells in the peripheral blood and detected by imaging flow cytometry. Data are presented as means ± SD of independent experiments. (**d**) Relative number of CTCs in 100,000 peripheral blood mononuclear cells in HCC patients with MVI (M1 & M2) and HCC patients without MVI (M0). CTCs were defined as CD45^−^ & EpCAM^+^ cells in peripheral blood and detected by imaging flow cytometry. Data are presented as means ± SD of independent experiments. *P < 0.05. (**e**) ROC curve of CTCs for 23 HCC patients with MVI (M1 & M2) and 18 HCC patients without MVI (M0) in 52 total samples. CTCs were defined as CD45^−^ & EpCAM^+^ cells in peripheral blood and detected by imaging flow cytometry.

To further analyze the use of HKR cells in peripheral blood samples for CTC detection, an ROC curve analysis was conducted. To predict the development of liver cancer, this method achieved a specificity and sensitivity of 1.00. Based on a Youden index analysis^7^, the optimal threshold was 21.8 HKR cells per 100,000 cells (Fig. 3a). To determine whether a HCC patient had MVI, the AUC was 0.8214 (Fig. 3b). According to the Youden index curve analysis, 57.3 HKR cells per 100,000 cells was the optimal threshold, the sensitivity was 85.19%, and the specificity was 78.35%. These results indicate that HKR detection can not only distinguish patients with cancer from healthy volunteers, but can also facilitate the diagnosis of MVI. To further assess the sensitivity of HKR cell detection based on imaging flow cytometry, traditional CTC detection based on CD45^−^EpCAM^+^ cells was performed using the same samples.

In this analysis, 4.7 ± 2.2 per 100,000 cells were CD45^−^ & EpCAM^+^ cells in the peripheral blood of cancer patients, and this number did not differ significantly from that of non-cancer patients (2.8 ± 1.5/100,000) (Fig. 3c and Supplementary Figure S4; *P* = 0.2075). In addition, 8.7 ± 4.2/100,000 CD45^−^ & EpCAM^+^ cells were detected in patients with MVI. This value was significantly different from that of patients without MVI (3.6 ± 1.5/100,000), but the difference was not as highly significant as that obtained using flow cytometry (Supplementary Figure S4 and Fig. 3d; *P* = 0.0377). An ROC curve analysis indicated that the AUC value for this method was 0.734, which was lower than that of the method based on the nuclear-cytoplasmic ratio (Fig. 3e).

### Association between HKR cell detection and prognosis

**Figure 4:**
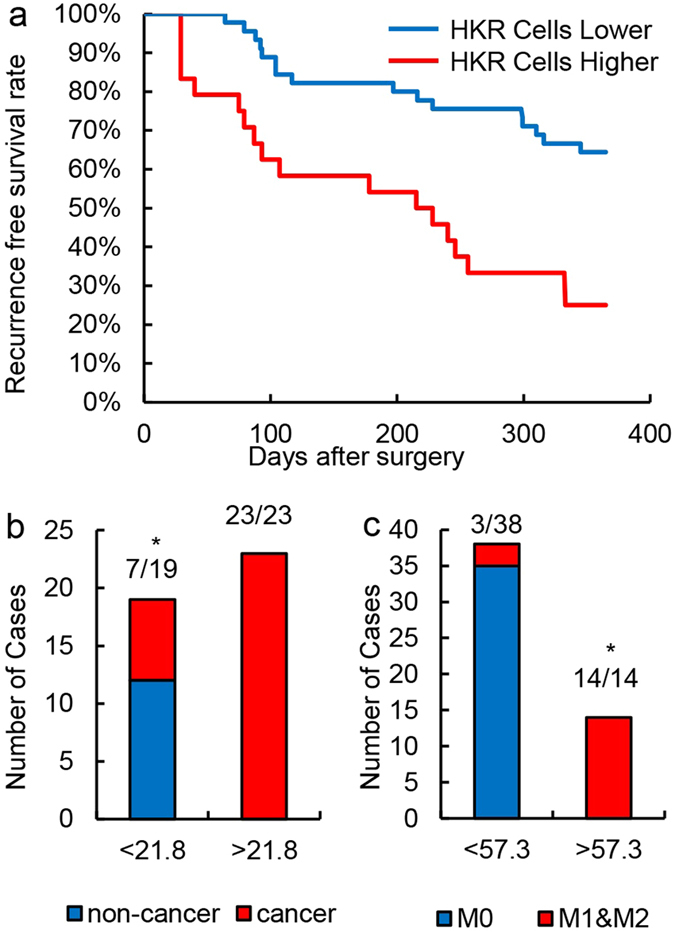
Association between prognosis and HKR cells. (**a**) Recurrence-free survival curve of HCC patients. Different patient survival rates between the high HKR cell group (HKR cells > 57.3/100,000 PBMC) and low HKR cell group are shown. (**b**) Number of cancer cases in the high and low HKR groups. HKR cells were detected by imaging flow cytometry. A threshold of 21.8/100,000 PBMCs was used. *P < 0.05 C: Number of MVI (M0 or M1&M2) cases (HKR cells > 57.3/100,000 PBMCs) in HKR and other groups. HKR cells were defined and detected by imaging flow cytometry. A threshold of 57.3/100,000 PBMCs was used.

To evaluate prognosis, the HCC test cohort was observed over a -month follow-up period. With the threshold of 57.3 HKR cells per 100,000 cells, 57 cancer patients were divided into two groups, including 12 cases of low HKR cell counts (<57.3/100,000 cells) and 45 cases of high HKR cell counts (>57.3/100,000 cells). During the follow-up period, 22/45 cases in the high HKR group exhibited recurrence and 6 patients died, while 3/12 cases in the low HKR group exhibited recurrence and no patients died (Fig. 4a). This indicates that this method can be used to predict prognosis in patients with high sensitivity, and specifically to estimate whether the patient will experience recurrence within a short period.

### HKR cell detection for prospective studies

To evaluate the accuracy of the method, another 42 random samples were obtained. Peripheral blood samples of these 42 patients were subjected to HKR cell detection. All samples were divided into groups according to HRK cell counts. When a threshold of 21.8/100,000 cells was used, all 23 samples that exceeded the threshold were confirmed as HCC (Fig. 4b). However, only 7 of 19 samples below the threshold were confirmed as cancer patients. Other samples were confirmed as non-cancer patients with liver disease (hepatitis B or cirrhosis) (Fig. 4b). When a threshold of 57.3/100,000 cells was used, all 14 samples that exceeded the threshold were confirmed as HCC with MVI (Fig. 4c). Only 3 of 38 samples below the threshold were confirmed as MVI (M1 & M2, Fig. 4c). These results indicate that the detection method based on HKR cells is not only useful for cancer diagnosis, but can also be used as a sensitive method for MVI diagnosis.

## Discussion

In this study, a CTC detection method based on imaging flow cytometry was developed. Rather than antibodies, this method was based on a morphological property shared by all tumor cells, i.e., a high karyoplasmic ratio. Using this method, we discovered significant relationships between HKR cell counts and MVI, which might facilitate pre-operative predictions of the presence of MVI. We obtained a high sensitivity for the diagnosis of MVI preoperatively, especially when the HKR cell counts were higher than 57.3 CTCs/100,000 cells.

MVI, also known as microvascular thrombosis, is a form of venous thrombosis affecting the portal venule or hepatic capillary. According to a recent study^9^, MVI is present in15.0–57.1% of liver resection specimens; it is an indicator of aggressive behavior and poor outcomes for HCC patients^10^.

Traditional methods for CTC detection in peripheral blood are based on CTC surface markers, such as CD133 and EpCAM^5^. However, even in tumor tissues, only up to 20% of cells express the three markers^6^. Furthermore, owing to the EMT effect, many tumor cells lose these surface markers as they are shed into circulating blood^11^. Accordingly, some assays based on cell surface markers can only detect a very small fraction of CTCs and cannot be used to obtain accurate estimates of CTCs. Owing to the complexity of HCC^12^, these surface marker-based methods are not effective for CTC detection. Our approach was based on morphological properties that are shared among all tumor cells, resulting in a high sensitivity. Thus, compared with previous methods that detect approximately 1–10/100,000 CTCs in the peripheral blood of cancer patients^13^, our method can detect several times more CTCs. Patients with HKR cell counts of greater than 57.3/100,000 cells belong to a high-risk subgroup for MVI (positive predictive value, 57.4%). Additionally, the fluorescence intensity of the nucleus did not contribute to the estimate of the CTC cell content because the DNA content of tumor cells is similar to that of normal cells. However, nuclei appear larger. As shown in Fig. 1d and f, normal cells and CTC cells differed significantly with respect to nucleus size, but did not differ in fluorescence intensity.

Another advantage of our method is its ease. Antibodies were not required, and only Red Blood Cell Lysis Buffer and DAPI staining solution were needed; accordingly, the method was economical. Furthermore, only 1 mL of peripheral whole blood from HCC patients was needed to establish a preoperative diagnosis of MVI. Therefore, this method is expected to be very safe, especially for patients who experience high blood loss during surgery. In addition, the practicality and stability of imaging flow cytometry are well-established^14^. This supports the credibility of results obtained using our method.

Despite its advantages, this method still has some limitations. This method relies on the karyoplasmic ratio to distinguish cancer cells from PBMCs. However, some immune cells, such as macrophages and basophilic pronormoblasts, also have a high karyoplasmic ratio. Therefore, the detection of CD45, the leukocyte common antigen, is essential, and can effectively reduce interference owing to immune diseases. The detection of cancer biomarkers, such as alpha-fetoprotein, can be used to pre-screen patients to enhance the detection accuracy.

Preoperative predictions based on our method can be used to select patients for randomized clinical trials to evaluate the efficacy of liver resection in patients with MVI or to recruit patients into studies of neoadjuvant therapies for HCC. These applications may optimize strategies to improve the outcomes of HCC patients with MVI. In addition, as the absence of MVI is essential in the new criteria for liver transplantation, the suitability of liver transplantation can be assessed without invasive examinations.

Our study was limited by the small sample size, i.e., 123 total patients and less than 20 patients in some groups. Additionally, the follow-up duration was only 12 months, which was insufficient. Finally, our research focused on hepatitis B liver cancer. In future studies, we will expand the sample size and extend the follow-up period. We will detect HKR cells in patients with HCV-HCC, prostate cancer, and lung cancer to verify the clinical applicability of the method.

## Conclusions

In this study, an HKR cell assay was developed for CTC detection based on imaging flow cytometry. The technique relies on the karyoplasmic ratio, rather than antibodies or cell surface markers, and has a higher sensitivity than those of traditional techniques, indicating that it is a more effective method for CTC detection and monitoring in HCC.

## Methods

All authors had access to the study data and reviewed and approved the final manuscript. All methods were performed in accordance with the relevant guidelines and local regulations.

### Patients and surgery

Consecutive patients with hepatitis B liver cancer or other non-cancer liver diseases and healthy controls were recruited from Eastern Hepatobiliary Surgery Hospital, Second Military Medical University, Shanghai, China, between September 29, 2015 and March 18, 2016. All patients were diagnosed based on both imaging examination and biochemical analyses, and were confirmed by histopathology, according to the American Association for the study of Liver Disease guidelines, especially hepatitis B liver cancer^15^. All liver resection specimens were examined histopathologically, with a focus on the detection of MVI. The examinations were performed independently by three pathologists.

The definition of MVI was the same as that reported by Roayaie *et al*.^9^. Briefly, MVI was defined as the presence of tumor cells in a portal vein, hepatic vein, or a large capsular vessel of the surrounding hepatic tissue lined by the endothelium that is visible only by microscopy. The MVI scores were based on the Evidence-based Practice Guidelines for the Standardized Pathological Diagnosis of Primary Liver Cancer^16^. Briefly, according to the number of invaded vessels, patients were classified into three groups as follows: M0, no evidence of MVI; M1, one to five invaded vessels; M2, more than five invaded vessels.

The diagnostic criteria for tumor recurrence were similar to those used for the initial HCC diagnosis; briefly, these criteria were as follows: the appearance of new lesions with typical radiological features of HCC in two imaging examinations^15^. The endpoints of this study were overall survival and time to recurrence. Overall survival was measured from the date of liver resection to the date of death or the date of the last follow-up visit. The time to recurrence was calculated from the date of liver resection to the date when tumor recurrence was diagnosed. The healthy controls were eligible blood donors with normal liver biochemistry and no history of liver disease.

On this basis, 12 normal individuals were included as the negative control. The volunteers had the same gender, race, and age composition as those of the experimental group (Supplementary Table 1). The number of HKR cells was detected in the negative control and used as a baseline value to evaluate the test group. 10 HBV-HCC patients with MVI and 5 normal individuals were included as the training set. The number of HKR cells was detected in the training set to evaluate the test method.

Data collection and analyses were performed by three independent researchers. The study was approved by the Institutional Ethics Committee of the Eastern Hepatobiliary Surgery Hospital. Written informed consent was obtained from all patients included in the study. Patients did not receive financial compensation. All methods were performed in accordance with the relevant guidelines and local regulations.

### Circulating tumor cell flow cytometry assay

Blood samples (5 mL) for the flow cytometry analysis were collected in conical 5-mL tubes with EDTA. Whole blood was added to cell preparation tubes and centrifuged for 25 min at 1500× *g* at room temperature. The upper phase of the separated plasma was removed and the lower phase was transferred to 50-mL centrifuge tubes. After fixation with Red Blood Cell Lysis Buffer (containing 155 mM NH_4_Cl, 10 mM NaHCO_3_, 100 mM EDTA, pH 7.4), all samples were washed twice with up to 50 mL of physiological saline and centrifuged at 1000× *g* for 7 min at 4 °C. The pellets were resuspended in phosphate-buffered saline.

After defrosting on ice, samples were washed twice with 1 mL of phosphate-buffered saline containing 2 mM EDTA and 0.5% weight/volume bovine serum albumin. After each wash step, samples were centrifuged at 1000× *g* for 4 min at room temperature. Next, the supernatant was removed and the pellets were resuspended in the remaining 100 mL of Flow Cytometry Staining Buffer (Cat. No. 00-4222; eBioscience, San Diego, CA, USA). EpCAM-FITC antibody (Cat. No.: 11-5791; eBioscience) was used for CTC staining and the CD45-PE-Cy5 antibody (Cat. No.: 15-0459-42; eBioscience) was used for leucocyte labeling. After the removal of unreacted antibodies, samples were quantified by fluorescence-activated cell sorting (FACS) and resuspended in DAPI staining buffer (containing 3 μM DAPI, 100 mM Tris, pH 7.4, 150 mM NaCl, 1 mM CaCl_2_, 0.5 mM MgCl_2_, and 0.1% NP-40) for 30 min.

The ImageStreamX FlowSight (Merck Millipore, Seattle, WA, USA) single camera system with four lasers was used for the imaging flow cytometry assay. The ImageStreamX FlowSight system was calibrated using calibration beads (Merck Millipore) before the test, and the sample data were acquired under laser excitation. Laser powers of 50 mW at 488 nm and 20 mW at 561 nm were used for the EpCAM and CD45 tests, and a laser power of 10 mW at 405 nm was used for the nuclear-cytoplasmic ratio test. Images (bright field in channel 1, FITC in channel 2, PECY5 in channel 5, and DAPI in channel 7) were acquired for each cell. The cells with cross-sectional areas of larger than 100 μm^2^ and positive DAPI signals were valid cells. A total of 100,000 individual valid cells were analyzed for each experimental sample. INSPIRE (version 6.0.154, Merck Millipore) was used for data collection.

### Imaging flow cytometry analysis

Raw image files (RIF format, Generated by INSPIRE software) were analyzed using algorithms in IDEAS (version 6.1.822.0, Merck Millipore) image analysis software. After data acquisition, single cells were analyzed based on area and aspect ratio parameters. For each cell, the bright field, FITC (Channel 2), PeCy5 (in Channel 5), and DAPI (in Channel 7) images were used for the analysis.

The signals from the bright field and DAPI channels were used for the masks of cells and their corresponding cytoplasm (Supplementary Figure S2). The ratio of the area of the two masks (DAPI area/Cell area) was defined as the karyoplasmic ratio.

The threshold and discrete values of the karyoplasmic ratio were obtained for normal human peripheral blood samples, and the number of abnormal cells in normal samples was used as the background value. The same method was applied to hepatic cancer specimens. The number of abnormal karyoplasmic ratios that exceeded the background value was defined as the number of high karyoplasmic ratio (HKR) cells. IDEAS was used to calculate the number of HKR cells and record the average and discrete karyoplasmic ratios. Imagines for all cells and signal channels were recorded for further analyses.

### Statistical analysis

The number of HKR cells in PBMCs was divided into 200 percentiles, and the sensitivity and specificity were calculated for each and combined to obtain the Youden index. These 200 Youden indexes were used to generate a Youden index curve, and the highest point indicated the HKR cell number with the highest diagnostic value. This HKR cell number was used as the optimal threshold value for the subjects.

Statistical analyses were implemented in SPSS for Windows (version 16.0). Differences between two independent groups were evaluated using Mann–Whitney U tests (for continuous variables and nonparametric analyses). Binomial Tests were performed for non-parametric test (Fig. 4b) using SPSS. To assess the sensitivity, specificity, and respective areas under the curve (AUCs) with 95% CIs for this method, receiver operating characteristic (ROC) curves were obtained. The optimum cutoff value used for diagnosis was investigated by maximizing the sum of the sensitivity and specificity and minimizing the overall error (square root of the sum [1 − sensitivity]^2^ + [1 − specificity]^2^), and by minimizing the distance from the cutoff value to the top-left corner of the ROC curve.

The correlations between the number of HKR cells in peripheral blood samples and the diagnosis of cancer or the presence of MVI were analyzed using Pearson’s χ^2^ tests or Fisher’s exact tests. *P*-values of less than 0.05 (two-sided) were considered significant.

## Additional Information

**How to cite this article**: Liu, Z. *et al*. Circulating tumor cell detection in hepatocellular carcinoma based on karyoplasmic ratios using imaging flow cytometry. *Sci. Rep.* **6**, 39808; doi: 10.1038/srep39808 (2016).

**Publisher's note:** Springer Nature remains neutral with regard to jurisdictional claims in published maps and institutional affiliations.

## Supplementary Material

Supplementary Information

## Figures and Tables

**Table 1:** Content, nuclear area, and DAPI intensity of HKR cells in 10 HCC patients with MVI and 5 healthy volunteers.

ID Number	Group	Cell Number	Normal cells			HKR cells		
			Count	Karyoplasmic Ratio	Intensity of DAPI	Count	Karyoplasmic Ratio	Intensity of DAPI
177675	MVI-HCC	97326	97257	0.471 ± 0.090	47237.9 ± 6736.4	69	0.853 ± 0.089	45699.1 ± 2902.6
177698	MVI-HCC	92716	92626	0.537 ± 0.140	45665.8 ± 9949.9	90	0.911 ± 0.081	43461.2 ± 2790.1
177719	MVI-HCC	98255	98207	0.569 ± 0.093	52870 ± 6222.3	48	0.814 ± 0.071	50888.9 ± 2791.2
177722	MVI-HCC	97572	97510	0.585 ± 0.107	53371.7 ± 7415.6	62	0.681 ± 0.059	50802.7 ± 2577.5
177727	MVI-HCC	93280	93202	0.563 ± 0.149	45861.8 ± 10266.1	78	0.889 ± 0.074	43503.7 ± 2663.2
177760	MVI-HCC	92722	92631	0.477 ± 0.120	45404.9 ± 9293.4	91	0.832 ± 0.063	42908.6 ± 3183.8
177761	MVI-HCC	92954	92885	0.472 ± 0.120	45564 ± 9481	69	0.721 ± 0.060	51112.3 ± 3120.4
177768	MVI-HCC	97512	97458	0.488 ± 0.090	52268.2 ± 7334.9	54	0.768 ± 0.059	49692.6 ± 3406.9
177781	MVI-HCC	92844	92791	0.598 ± 0.110	53371.7 ± 7415.6	53	0.863 ± 0.075	57770.1 ± 3322.5
177784	MVI-HCC	94583	94516	0.521 ± 0.138	45861.8 ± 10266.1	67	0.870 ± 0.073	57585.2 ± 2880.5
137781	Healthy	100000	99993	0.580 ± 0.116	48441.3 ± 7759.6	7	0.891 ± 0.075	54973.5 ± 4066.7
137784	Healthy	100000	99994	0.487 ± 0.103	52829.1 ± 9017.9	6	0.834 ± 0.067	54070.7 ± 4076.7
137788	Healthy	82976	82970	0.501 ± 0.107	52051.2 ± 9265.7	6	0.807 ± 0.072	48806.4 ± 3923.6
137865	Healthy	100000	99989	0.473 ± 0.144	46047 ± 6648.2	11	0.939 ± 0.130	34591.2 ± 3036.9
137872	Healthy	100000	99994	0.536 ± 0.128	44805.8 ± 8590.2	6	0.727 ± 0.059	59536.4 ± 2825

Karyoplasmic ratio = DAPI area/Total cell area. Data are presented as the mean ± SD of independent experiments.

**Table 2:** The source or composition of the samples and average contents of HKR cells.

Diagnosis	Number	HKR cells
Health	12	6.8 ± 3.7
Cancer	57	56.2 ± 23.8
Cholangiocarcinoma	5	58.2 ± 26.8
HCC	52	42.0 ± 15.3
M0	18	32.2 ± 11.6
M1	18	55.3 ± 19.9
M2	16	59.3 ± 27.8
Non-cancer	12	7.6 ± 1.2
Hepatitis B	2	8.0 ± 1.4
Hemangioma	2	6.2 ± 0.2
Choledochocyst	2	5.6 ± 2.9
Focal nodular hyperplasia	2	10.7 ± 2.0
Hepatic calculus	4	7.5 ± 1.8

The data are presented as means ± SD of independent experiments.

**Table 3:** Clinical characteristics of all samples (including 52 HCC patients and other control group) and their HKR cell contents.

Variable	Number of Patients	HKR cells content per 100,000 cells	P-value (χ^2^)
Cancer or non-Cancer			2.29E-10
Cancer	57	56.2 ± 23.8	
Non-cancer	12	7.6 ± 1.2	
Cancer source			0.0544337
HCC	52	42.0 ± 15.3	
Cholangiocarcinoma	5	58.2 ± 26.8	
Metastasis			0.002075
Found	18	84.5 ± 23.8	
No-found	39	39.5 ± 13.2	
Organs Invasion			0.040694
Found	14	87.5 ± 36.0	
No-Found	43	43.0 ± 15.6	
Microvascular invasion			0.000538
M0	18	32.2 ± 11.6	
M1&M2	34	62.0 ± 16.6	
Gender			0.382935
Male	50	50.7 ± 24.0	
Female	29	39.9 ± 22.2	
HBV			0.494083
≤1000 copy/mL	24	48.9 ± 28.0	
>1000 copy/mL	55	41.6 ± 13.6	
Ascites			0.239173
Yes	14	65.9 ± 12.27	
No	55	49.6 ± 23.3	
Total bilirubin (STB)			0.10713
≤17.1 μmol/L	23	55.7 ± 25.6	
>17.1 μmol/L	46	37.7 ± 15.8	
Albumin (Alb)			0.879635
≤35 g/L	27	50.4 ± 23.9	
>35 g/L	42	52.4 ± 23.8	
Prothrombin time (PT)			0.754196
<10 s	28	48.0 ± 24.2	
>10 s	41	51.9 ± 20.5	
Capsule			0.146648
No-capsule	32	54.6 ± 12.5	
Capsule	15	42.0 ± 15.33	

The data are presented as means ± SD of independent experiments.
