# Supplementary material for: Circulating tumor cell detection in hepatocellular carcinoma based on karyoplasmic ratios using imaging flow cytometry
Source: Sci Rep. 2016 Dec 23;6:39808. doi: 10.1038/srep39808 (PMC5180239; doi:10.1038/srep39808)
Supplement: Supplementary Information [file srep39808-s1.pdf]

Circulating tumor cell detection in hepatocellular carcinoma based on karyoplasmic ratios using imaging flow cytometry

Zixin Liu#1, Weixing Guo#1, Dandan Zhang#2, Yanan Pang2, Jie Shi1, Siqin Wan1, Kai Cheng2, Jiaqi Wang*2, Shuqun Cheng*1

1. Eastern Hepatobiliary Surgery Hospital (EHBH), the Second Military Medical University, Shanghai 200433, China.

2. Clinical Research Center, Changhai Hospital, Second Military Medical University, Shanghai 200433, China

#These authors are co-first authors and contributed equally to this work.

*These authors are the corresponding authors of this work.

Correspondence：

Professor Shuqun Cheng at Eastern Hepatobiliary Surgery Hospital, Second Military Medical University, 225 Changhai Road, Shanghai, China. Telephone: (86)21-81875251. Fax: (86)21-65562400, E-mail: chengshuqun@aliyun.com.

Professor Jiaqi Wang, Clinical Research Center, Changhai Hospital, Second Military Medical University, Shanghai, 168 Changhai Road 200433, China. Telephone: +86-21-31162097; Fax: +86-21-31162091; E-mail: darkmk2@163.com.

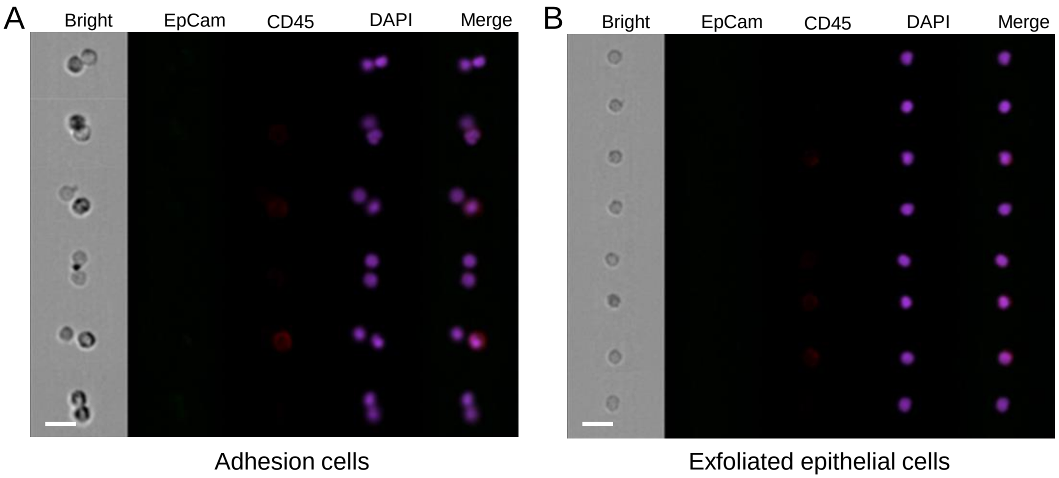

Supplementary Figure 1

Representative results of the cell images in PBMC of HCC patients.

A: The Image of Adhesion cells
B: The Image of Exfoliated epithelial cells
FITC: EpCam PE-Cy5: CD45 DAPI: Cell nucleus
Scale bars represent 20 μm

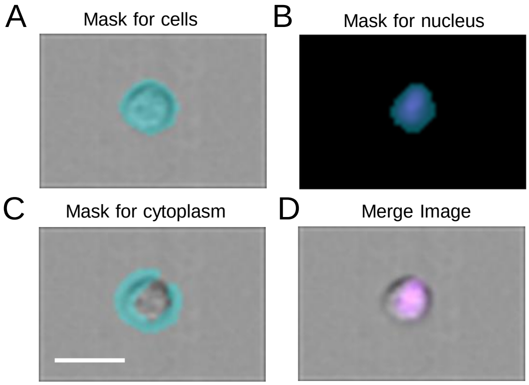

Supplementary Figure 2

The Output Mask for Cell Image

A: Output mask for cells
B: Output mask for cell nucleus
C: Output mask for cytoplasm
D: Merge Image for cells
Scale bars represent 20 μm

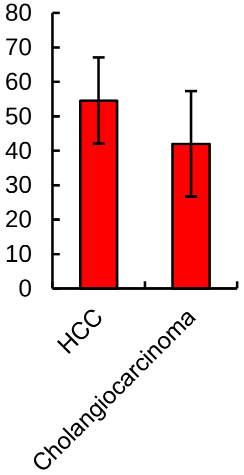

Supplementary Figure 3

Relative content of HKR cells in HCC group and Cholangiocarcinoma group

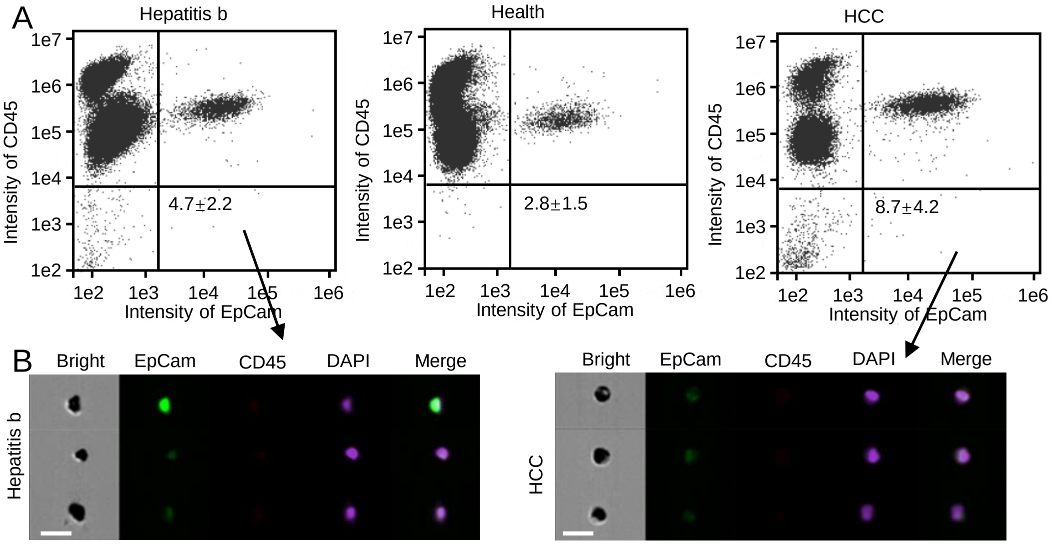

Supplementary Figure 4

The image flow cytometry test results for CTCs

A: The flow cytometry test results for CD45&EpCam in Hepatitis b patients, HCC patients and healthy volunteers

B: The Image of CD45-and Epcam+ cells.

Scale bars represent 20 μm

.

Diagnosis	Cancer		Non-Cancer	Healthy
HKR cells	<57.3	>57.3		
Sex	45	12		
Male	36 (80%)	9 (75%)	5 (42%)	8 (67%)
Female	9 (20%)	3 (25%)	7 (58%)	4 (33%)
Age	53.8±12.1	54.4±10.2	46.5±17.6	51.7±7.5
AFP	310±483	305±470	1.9±0.3	4.8±2.6
HBV DNA				
>50IU/ml	25 (56%)	6 (50%)	3 (25%)	0 (0%)
<50IU/ml	20 (44%)	6 (50%)	9 (75%)	12 (100%)
Ascites	2 (4%)	2 (17%)		
T bilirubin	19.2±31.3	20.2±34.0	15.0±10.3	7.9±3.5
Albumin	41.4±4.0	40.0±7.2	41.6±4.3	42.5±4.2
Thrombinogen	11.5±1.6	11.1±1.9	11.4±2.1	11.7±1.0
Tumor Number				
Single	11 (24%)	5 (42%)		
Multiple	34 (76%)	7 (58%)		
MVI				
M0	22 (49%)	3 (25%)		
M1	12 (27%)	3 (25%)		
M2	10 (24%)	6 (50%)		
Capsule				
No-Capsule	17 (38%)	7 (58%)		
Capsule	28 (62%)	5 (42%)		
Recurrence	21 (47%)	9 (75%)		
Death	2 (5%)	2 (17%)		

Supplementary Table 1

The clinical features of samples
